# A comparison between the clinical frailty scale and the hospital frailty risk score to risk stratify older people with emergency care needs

**DOI:** 10.1186/s12873-022-00730-5

**Published:** 2022-10-25

**Authors:** Abdullah Alshibani, Tim Coats, Laia Maynou, Fiona Lecky, Jay Banerjee, Simon Conroy

**Affiliations:** 1 Department of Health Sciences, College of Life Sciences, University of Leicester, Leicester, LE1 7HA UK; 2 Emergency Medical Services Department, College of Applied Medical Sciences, King Saud Bin Abdulaziz University for Health Sciences, Riyadh, Saudi Arabia; 3 King Abdullah International Medical Research Center, Riyadh, Saudi Arabia; 4 University Hospitals of Leicester NHS Trust, Leicester, UK; 5 Department of Cardiovascular Sciences, Emergency Medicine Academic Group, University of Leicester, Leicester, UK; 6 Department of Health Policy, London School of Economics and Political Science, London, UK; 7 Department of Economics, Econometrics and Applied Economics, Universitat de Barcelona, Barcelona, Spain; 8 Center for Research in Health and Economics (CRES), Universitat Pompeu Fabra, Barcelona, Spain; 9 Centre for Urgent and Emergency Care Research, University of Sheffield, Sheffield, UK; 10 MRC Unit for Lifelong Health and Ageing, University College London, London, UK

**Keywords:** Geriatric, Elderly, Urgent Care, Frailty, Correlation, Outcome

## Abstract

**Background:**

Older adults living with frailty who require treatment in hospitals are increasingly seen in the Emergency Departments (EDs). One quick and simple frailty assessment tool—the Clinical Frailty Scale (CFS)—has been embedded in many EDs in the United Kingdom (UK). However, it carries time/training and cost burden and has significant missing data. The Hospital Frailty Risk Score (HFRS) can be automated and has the potential to reduce costs and increase data availability, but has not been tested for predictive accuracy in the ED. The aim of this study is to assess the correlation between and the ability of the CFS at the ED and HFRS to predict hospital-related outcomes.

**Methods:**

This is a retrospective cohort study using data from Leicester Royal Infirmary hospital during the period from 01/10/2017 to 30/09/2019. We included individuals aged + 75 years as the HFRS has been only validated for this population. We assessed the correlation between the CFS and HFRS using Pearson’s correlation coefficient for the continuous scores and weighted kappa scores for the categorised scores. We developed logistic regression models (unadjusted and adjusted) to estimate Odds Ratios (ORs) and Confidence Intervals (CIs), so we can assess the ability of the CFS and HFRS to predict 30-day mortality, Length of Stay (LOS) > 10 days, and 30-day readmission.

**Results:**

Twelve thousand two hundred thirty seven individuals met the inclusion criteria. The mean age was 84.6 years (SD 5.9) and 7,074 (57.8%) were females. Between the CFS and HFRS, the Pearson correlation coefficient was 0.36 and weighted kappa score was 0.15. When comparing the highest frailty categories to the lowest frailty category within each frailty score, the ORs for 30-day mortality, LOS > 10 days, and 30-day readmission using the CFS were 2.26, 1.36, and 1.64 and for the HFRS 2.16, 7.68, and 1.19.

**Conclusion:**

The CFS collected at the ED and the HFRS had low/slight agreement. Both frailty scores were shown to be predictors of adverse outcomes. More research is needed to assess the use of historic HFRS in the ED.

**Supplementary Information:**

The online version contains supplementary material available at 10.1186/s12873-022-00730-5.

## Introduction

Population ageing has significant implications for healthcare as increasing numbers of older patients living with frailty require hospital treatment—presenting often to Emergency Departments (EDs) [1]. Frailty is a state of increased vulnerability as a result of decreased reserve and function of multiple body systems with age which compromise the ability to cope with acute stressors. Several systematic reviews, looking at different patient populations, showed that frailty is significantly associated with adverse outcomes including high rates of short- and long- term mortality, complications, readmission, and increased Length of Stay (LOS), as well as adverse patient outcomes including functional decline and reduced quality of life related to physical function and mental health [2–8].

For patients attending EDs, identifying frailty was shown to have some advantages which included:

1. Prompting a more holistic approach for assessment.
2. Influencing clinical decision making and aiding in determining the appropriate approach for managing patients, specifically curative or palliative care.
3. Guiding disposition decisions of individuals attending in the ED or guiding referral to geriatric services.
4. Guiding service design through measuring the nature and magnitude of frailty, and mapping patient pathways [9].

Several frailty assessment tools have been assessed for use in the ED [10]. One of the widely used in such setting is the Clinical Frailty Score (CFS) which is accurate, practical, and rapid frailty assessment tool in the ED [11]. The CFS could predict adverse outcomes for admitted older people in the United Kingdom (UK) [12]. It has being embedded in many EDs in the UK.

The Hospital Frailty Risk Score (HFRS) is another example of a frailty risk assessment tool [13]. The HFRS has been shown to be able to identify patients with greater risk of adverse outcomes with low-cost and a systematic approach to the assessment of frailty [13]. However, The HFRS has only been validated in general medical inpatients, not specifically ED attenders.

The CFS is relatively quick, simple, and easy to use, but has a time/training cost and significant missing data. For example, previous evidence showed the CFS at the ED obtained from routinely collected data is dependent on routine clinical practice and, therefore, was shown to be associated with significant missing data although frailty assessment with the CFS has been implemented in the ED for several years [14]. The HFRS can be automated and has the potential to reduce costs and increase data availability, but has not been tested for predictive accuracy in the ED. If clinicians are to use (historical) HFRS scores (i.e., International Classification of Diseases 10^th^ edition [ICD–10] codes from previous two-year admissions prior current admission) in place of incident CFS scores, they need to be clear about its validity in this population and setting.

This study, therefore, aims to assess the correlation between and the ability of the CFS and HFRS to predict adverse hospital-related outcomes for older patients attending the ED. It is the first study, up to our knowledge, to address this topic for individuals attending at the ED. It will inform clinical practice and research about frailty assessment and the use of frailty assessment tools in the ED and provide several recommendations.

## Methods

We undertook a retrospective cohort study examining the correlation and the ability of the CFS and HFRS to predict adverse outcomes captured on routine data sets from a single centre. Descriptive analysis was performed to assess baseline characteristics. Pearson’s correlation coefficient and weighted kappa scores were applied to assess the correlation between the CFS and HFRS. Logistic regression models were performed to assess the ability of the CFS and HFRS to predict adverse outcomes. There was no follow-up for patients if admitted elsewhere outside the trust.

### Study setting

The study was carried out in a large single-site ED in the UK—Leicester Royal Infirmary (LRI) hospital. The estimated catchment population in Leicester is around 1.1 million people and nearly 165,000 are aged ≥ 65 years. More than 230,000 people attend at LRI ED each year, of whom around 48,000 are older adults.

Frailty assessment and screening has been implemented at LRI ED since 2016 [9]. It started with determining the most useful frailty assessment tool and then embedding the CFS scoring in the ED [9].

The hospital patient administration systems data contain a list of ICD-10 codes, which were used to construct the HFRS. The ICD-10 codes only calculated for hospital admissions, so only individuals attending in the ED who were admitted have HFRS available.

### Participants selection

This study included older individuals who were registered after their index ED presentation (i.e., first attendance in the ED) at LRI from 01/10/2017 to 30/09/2019, were admitted to hospital, and had their CFS at the ED and current HFRS scores after hospital admission. We used the age cut-off 75 + years in the inclusion criteria as the HFRS has been only validated to use for this population [13].

Given the exploratory nature of this study, no prior sample size calculation was undertaken. The LRI ED is known to have thousands of attendees every year (with high readmission and mortality rates expected in this population) – sufficient to perform the correlational and regression analysis.

### Methods of measurement

#### Clinical frailty score

The CFS is a 9-point scale which represents different levels of frailty severity [15]. The score of 1 represents very fit and, from this point, the frailty severity increases to a score of 8 (very severely frail), and 9 (terminally ill). For the purposes of this study, we assigned the different categories into four groups:

1. Non-frail (CFS 1–3),
2. Mild frailty (CFS 4–5),
3. Moderate frailty (CFS 6),
4. Severe frailty (CFS 7–9).

The CFS in this study was collected during the initial assessment of patients at the ED, typically by a triage-trained staff nurse or emergency physician, and based on the patients’ level of frailty two weeks prior to presentation.

#### Hospital frailty risk score

The HFRS is a frailty risk score which can be calculated based on ICD-10 diagnosis codes recorded in the patient’s index emergency admission [13]. All 20 diagnostic fields in all episodes are searched for 109 three-character ICD-10 codes included in the scoring algorithm, weighted points awarded for each code present and added together to create the final score [13]. Three risk categories can be created using cut-points which discriminated between individuals with different risks of adverse outcomes:

1. Low-risk of frailty (HFRS < 5).
2. Intermediate-risk of frailty (HFRS 5–15).
3. High-risk of frailty (HFRS > 15).

### Baseline covariates and outcome data

The assessed baseline and outcome data related to the index ED presentation of the study population. Baseline data included age, sex, CFS, acuity (National Early Warning Score [NEWS] – 2) [16], the Dynamic Priority Score (DPS) [17], and the Charlson Comorbidity Index (CCI) [18]. The DPS is a triage tool that is applied upon arrival at the ED to assess which individuals need more urgent care [17]. It was applied as a response to raised concerned in late 2015 in LRI hospital about the timeliness in which individuals arriving to the department by ambulance were assessed and establish their level of priority [17]. More information about the DPS and all other covariates is available in supplementary table 1.

#### Outcome Data

Subsequent hospital use and hospital-related outcomes were tracked for up to two years after index ED presentation. The follow-up period for the individuals in this study varied as they entered the cohort at different times. The outcomes were limited to service use outcomes (i.e., LOS and readmission), and mortality (in and out of hospital). All individuals were followed up until the study end, so no outcome data were lost.

### Primary data analysis

Baseline characteristics were reported with descriptive statistics; frequency and percentage for categorical variables, and mean (Standard Deviation [SD]) were reported for continuous variables. Outcomes were described in frequency and percentage by overall CFS or HFRS scores and then stratified by CFS and HFRS categorisations.

**Fig. 1:**
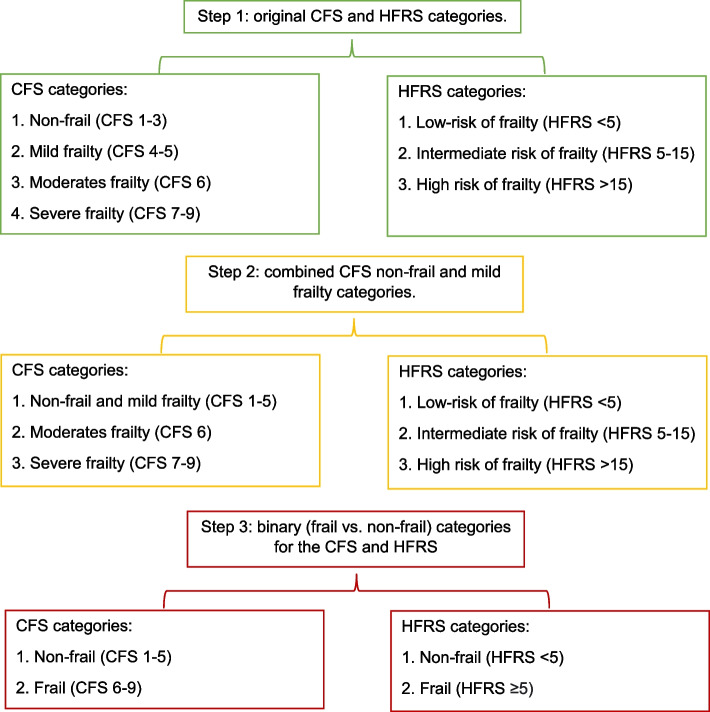
Steps of the kappa analysis in the study

To determine the correlation and level of agreement between the CFS and HFRS, we assessed the correlation of the continuous scores using Pearson’s correlation coefficient and categorised scores using three-steps weighted kappa scores (Fig. 1).

We also tested and compared the ability of the CFS and HFRS to predict adverse outcomes using binary logistic regression models for 30-day mortality (in or out of hospital), LOS > 10 days, and 30-day readmission.

We performed a univariate analysis for baseline covariates (age, sex, CCI, NEWS-2, and DPS). We predetermined if any of these covariates were statistically significant (*p* < 0.001) or clinically important for predicting any outcome, they would be accounted for within the multivariate logistic regression models. Due the non-hierarchical nature of the DPS score along with having only five individuals who were classified as standard in this study, we rearranged it to be more clinically ordered from low to more urgency level as follows: 1) standard + urgent, 2) very urgent, and 3) immediate resuscitation. We also performed the univariate and multivariate logistic regression analyses for both the CFS (by original categorisation and by combining non-frail and mild frailty categories) and the HFRS. The results were shown in Odds Ratios (ORs), 95% Confidence Intervals (CIs), and p-values. All analyses were performed in Stata (version 16; StataCorp, College Station, TX).

The study was undertaken as a service evaluation under the auspices of the University Hospitals of Leicester frailty strategy, so no ethical approval was required. Governance approvals were granted by the hospital’s Clinical Audit and Service Evaluation department.

## Results

### Characteristics of the study subjects

#### Study subjects

**Fig. 2:**
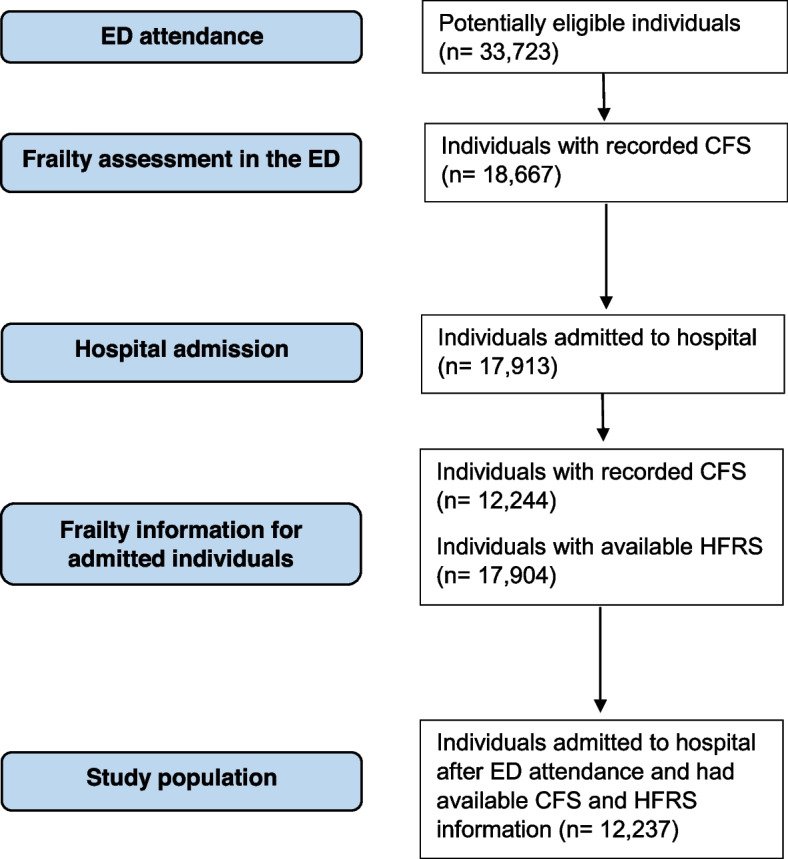
Flowchart of the study population

We obtained data on 33,723 potentially eligible individuals representing 87,361 ED attendances within the study period (Fig. 2). After applying our inclusion criteria, a total of 12,237 individuals were included. The mean age for these individuals was 84.6 years (SD 5.9) and 7,074 (57.8%) were females. The baseline characteristics for potentially eligible and included individuals are available in Table 1.

**Table 1:** Baseline characteristics of potentially eligible population and the study population stratified by their CFS and HFRS categories

Variable	Potentially eligible population				Study population							
	**Overall** **(*n* = 33,723)**	**With recorded CFS at ED presentation** **(*n* = 18,667)**	**With recorded CFS and admitted to hospital** **(*n* = 12,244)**	**With available HFRS** **(*n* = 17,904)**	**With both CFS + HFRS** **(*n* = 12,237)**	**CFS score**				**HFRS score**		
						**1–3** **(*n* = 2,139)**	**4–5** **(*n* = 5,195)**	**6** **(*n* = 2,939)**	**7–9** **(*n* = 1,964)**	** < 5** **(*n* = 4,227)**	**5–15** **(*n* = 5,866)**	**> 15** **(*n* = 2,144)**
**Age, y**												
Missing, n	245	11	1	7	1	0	0	1	0	0	1	0
Mean (SD)	83.6 (5.8)	84.3 (5.9)	84.6 (5.9)	84.1 (5.8)	84.6 (5.9)	81.6 (4.9)	84.2 (5.5)	86.2 (5.9)	86.3 (6.1)	83.1 (5.6)	85.0 (5.8)	86.2 (5.8)
**Female Sex, n (%)**	19,529 (57.9%)	11,102 (59.5%)	7,078 (57.8%)	10,122 (56.5%)	7,074 (57.8%)	1,039 (48.6%)	2,971 (57.2%)	1,828 (62.2%)	1,236 (62.9%)	2,297 (54.3%)	3,468 (59.1%)	1,309 (61.1%)
**CCI (calculated only when there was a hospital admission), n (%)**												
Admitted	17,913 (53.1%)	12,244 (65.6%)	12,244 (100%)	17,904 (100%)	12,237 (100%)	2,139 (100%)	5,195 (100%)	2,939 (100%)	1,964 (100%)	4,227 (100%)	5,866 (100%)	2,144 (100%)
0	5,423 (30.3%)	3,628 (29.6%)	3,628 (29.6%)	5,414 (30.2)	3,621 (29.6%)	975 (45.6%)	1,638 (31.5%)	670 (22.8%)	338 (17.2%)	1,725 (40.8%)	1,618 (27.6%)	278 (13.0%)
1–2	8,378 (46.8%)	5,844 (47.7%)	5,844 (47.7%)	8,378 (46.8%)	5,844 (47.8%)	868 (40.6%)	2,456 (47.3%)	1,488 (50.6%)	1,032 (52.6%)	1,893 (44.8%)	2,853 (48.6%)	1,098 (51.2%)
3–5	3,313 (18.5%)	2,229 (18.2%)	2,229 (18.2%)	3,313 (18.5%)	2,229 (18.2%)	222 (10.4%)	865 (16.7%)	664 (22.6%)	478 (24.3%)	425 (10.1%)	1,136 (19.4%)	668 (31.2%)
≥ 6	799 (4.5%)	543 (4.4%)	543 (4.4%)	799 (4.5%)	543 (4.4%)	74 (3.5%)	236 (4.5%)	117 (4.0%)	116 (5.9%)	184 (4.4%)	259 (4.4%)	100 (4.7%)
**National Early Warning Score—2**												
Recorded, n (%)	27,837 (82.5%)	16,757 (89.8%)	11,441 (93.4%)	16,348 (91.3%)	11,434 (93.4%)	1,975 (92.3%)	4,835 (93.1%)	2,752 (93.6%)	1,872 (95.3%)	3,936 (93.1%)	5,491 (93.6%)	2,007 (93.6%)
Mean (SD)	1.8 (2.5)	1.9 (2.5)	2.3 (2.7)	2.4 (2.8)	2.3 (2.7)	1.7 (2.3)	2.0 (2.4)	2.5 (2.7)	3.4 (3.3)	2.1 (2.5)	2.4 (2.8)	2.4 (2.8)
**DPS, n (%)**												
Recorded	33,696 (99.9%)	18,666 (≈ 100%)	12,244 (100%)	17,898 (≈ 100%)	12,237 (100%)	2,139 (100%)	5,195 (100%)	2,939 (100%)	1,964 (100%)	4,227 (100%)	5,866 (100%)	2,144 (100%)
Standard	26 (0.1%)	9 (0.1%)	5 (0.04%)	7 (0.04%)	5 (0.04%)	0	2 (0.04%)	2 (0.1%)	1 (0.1%)	2 (0.1%)	2 (0.03%)	1 (0.1%)
Urgent	21,935 (65.1%)	12,204 (65.4%)	7,169 (58.6%)	9,801 (54.8%)	7,164 (58.5%)	1,219 (57.0%)	3,187 (61.4%)	1,781 (60.6%)	977 (49.8%)	2,421 (57.3%)	3,455 (58.9%)	1,288 (60.1%)
Very urgent	7,277 (21.6%)	4,272 (22.9%)	3,127 (25.5%)	4,523 (25.3%)	3,126 (25.6%)	620 (29.0%)	1,328 (25.6%)	692 (23.6%)	486 (24.8%)	1,199 (28.4%)	1,412 (24.1%)	515 (24.0%)
Immediate Resuscitation	4,458 (13.2%)	2,181 (11.7%)	1,943 (15.9%)	3,567 (19.9%)	1,942 (15.9%)	300 (14.0%)	678 (13.1%)	464 (15.8%)	500 (25.5%)	605 (14.3%)	997 (17.0%)	340 (15.9%)

#### Frailty and baseline information

The mean age of the individuals increased as CFS and HFRS frailty severity increased (Table 1). The proportion of women also seemed to increase with increasing CFS and HFRS frailty severity (Table 1). The proportions of individuals with categorised CFS and HFRS are available in Table 1.

Individuals with CFS severe frailty, compared to those with HFRS high risk of frailty, had higher proportion of the highest CCI category (CCI ≥ 6) (5.9% vs. 4.7%) and higher NEWS-2 mean in the ED (NEWS-2 mean [SD], 3.4 [3.3] vs. 2.4 [2.8]). The proportion of DPS immediate resuscitation increased as CFS frailty severity increased, which was not the case with increasing HFRS where individuals with intermediate risk of frailty had the highest proportion than HFRS high risk of frailty (Table 1).

### Correlation between the CFS and HFRS

Overall, the Pearson’s correlation coefficient for the continuous CFS and HFRS was 0.36 (95% CI 0.34 – 0.38). The agreement between the two categorised CFS and HFRS when using original categories (supplementary table 2) showed low/slight agreement (weighted kappa of 0.10 (95% CI 0.09 – 0.11)).

Table 2, with a combined CFS non-frail & mild frailty category, was generated. Table 2 showed low/slight agreement between the CFS and HFRS — relatively large numbers of individuals is in the highest category for one measure but is the lowest for the other. Indeed, the weighted Kappa scores for these categories was 0.15 (95% CI 0.14 – 0.16).

**Table 2:** Two way cross-tabulation of CFS and HFRS categories, with combined CFS non-frail & mild frailty

		**CFS**			**Total**
		**Non- frail & mild (1–5)**	**Moderate (6)**	**Severe (7–9)**	
**HFRS**	**Low-risk (< 5)**	3,322 (27.1%)	600 (4.9%)	305 (2.5%)	4,227 (34.5%)
	**Intermediate-risk (5–15)**	3,236 (26.4%)	1,553 (12.7%)	1,077 (8.8%)	5,866 (47.9%)
	**High-risk (> 15)**	776 (6.3%)	786 (6.4%)	582 (4.8%)	2,144 (17.5%)
**Total**		7,334 (59.9%)	2,939 (24.0%)	1,964 (16.0%)	12,237 (100%)

When frailty was categorised into a binary measure (the CFS non-frail & mild frailty category matched to HFRS low-risk frailty and CFS intermediate & severe frailty were matched to HFRS intermediate and high-risk frailty) (supplementary table 3), the agreement level was fair (weighted kappa of 0.24 (95% CI 0.23 – 0.26)).

### Outcomes

Frailty categories with associated outcomes are presented in supplementary table 4. Individuals who were classified in the highest category from the CFS, compared to those classified from the HFRS, had higher proportions of 30-day mortality rate (25.6% vs. 19.5%) and 30-day readmission (36.7% vs. 28.8), but not LOS > 10 days (32.9% vs. 54.0%).

#### Baseline covariates and outcomes

In the univariate logistic regression analysis, age and CCI were significant predictors for 30-day mortality, LOS > 10 days, and 30-day readmission (Table 3). Male sex, NEWS-2, and DPS were significant predictors of 30-day mortality and 30-day readmission, but not for LOS > 10 days (Table 3). All of these covariates, therefore, entered the multivariate logistic regression. Additional information about the results of these covariates in the multivariate logistic regression models for both the CFS and HFRS is available in supplementary table 5.

**Table 3:** Univariate logistic regression for potential covariates

	**30-day mortality OR (95% CI)**	***P*-value**	**LOS > 10, OR (95% CI)**	***P*-value**	**30- readmission, OR (95% CI)**	***P*-value**
**Age**	1.04 (1.03 – 1.05)	< 0.001	1.02 (1.02 – 1.03)	< 0.001	1.02 (1.01 – 1.03)	< 0.001
**Sex**						
Female	Reference		Reference		Reference	
Male	1.21 (1.09 – 1.35)	< 0.001	0.89 (0.81 – 0.96)	0.002	1.20 (1.11 – 1.30)	< 0.001
**CCI**						
0	Reference		Reference		Reference	
1–2	1.72 (1.49 – 1.99)	< 0.001	1.54 (1.39 – 1.70)	< 0.001	1.26 (1.14 – 1.40)	< 0.001
3–5	2.92 (2.48 – 3.43)	< 0.001	2.03 (1.80 – 2.28)	< 0.001	1.74 (1.55 – 1.97)	< 0.001
≥ 6	6.56 (5.29 – 8.12)	< 0.001	2.34 (1.93 – 2.84)	< 0.001	3.54 (2.94 – 4.27)	< 0.001
**EWS**	1.20 (1.18 – 1.22)	< 0.001	1.02 (1.01 – 1.04)	0.002	1.12 (1.11 – 1.14)	< 0.001
**DPS**						
Standard and urgent	Reference		Reference		Reference	
Very urgent	1.48 (1.31 – 1.68)	< 0.001	1.06 (0.97 – 1.17)	0.211	1.30 (1.18 – 1.42)	< 0.001
Immediate Resuscitation	2.96 (2.60 – 3.36)	< 0.001	0.98 (0.88 – 1.10)	0.743	1.90 (1.70 – 2.11)	< 0.001

#### Clinical frailty score and outcomes

In the regression analysis, individuals with CFS mild, intermediate and severe frailty, when compared CFS non-frail individuals, had significantly higher 30-day mortality rate, LOS > 10 day, and 30-day readmission rate (supplementary table 6). However, after adjustment on covariates, 30-day mortality was significantly higher for moderate and severe frailty (OR (95% CI), 1.59 (1.29 – 1.97) and 2.66 (2.14 – 3.30), respectively) but not for mild frailty (OR (95% CI), 1.23 (1.00 – 1.50)) (*p* = 0.045), which was also the same for 30-day readmission (OR (95% CI), 1.34 (1.15 – 1.55) and 1.87 (1.60 – 2.19), respectively) versus (OR (95% CI), 1.18 (1.04 – 1.36)) (*p* = 0.013) (Table 4). After adjustment, LOS > 10 days was still significantly higher for individuals with mild, moderate and severe frailty (OR (95% CI), 1.57 (1.37 – 1.80), 1.97 (1.69 – 2.28), and 1.93 (1.64 – 2.27), respectively) (Table 4).

**Table 4:** Adjusted logistic regression for the CFS and HFRS*

Outcome	CFS categories (by original categories)	Adjusted OR (95% CI)	CFS categories (by combined non-frail and mild frailty categories)	Adjusted OR (95% CI)	HFRS categories (by original categories)	Adjusted OR (95% CI)
**30-day mortality**	Non-frail (1–3)	1.00	Non & mild frailty (1–5)	1.00	Low risk frailty (< 5)	1.00
	Mild frailty (4–5)**	1.23 (1.00 – 1.50) *p* = 0.045	Moderate frailty (6)	1.36 (1.18 – 1.56)	Intermediate risk frailty (15–15)	1.72 (1.49 – 1.99)
	Moderate frailty (6)	1.59 (1.29 – 1.97)	Severe frailty (7–9)	2.26 (1.96 – 2.61)	High risk frailty (> 15)	2.16 (1.82 – 2.56)
	Severe frailty (7–9)	2.66 (2.14 – 3.30)				
**LOS > 10 days**	Non-frail (1–3)	1.00	Non & mild frailty (1–5)	1.00	Low risk frailty (< 5)	1.00
	Mild frailty (4–5)	1.57 (1.37 – 1.80)	Moderate frailty (6)	1.39 (1.25 – 1.53)	Intermediate risk frailty (15–15)	2.73 (2.44 – 3.06)
	Moderate frailty (6)	1.97 (1.69 – 2.28)	Severe frailty (7–9)	1.36 (1.21 – 1.53)	High risk frailty (> 15)	7.68 (6.71 – 8.80)
	Severe frailty (7–9)	1.93 (1.64 – 2.27)				
**30-day emergency readmission**	Non-frail (1–3)	1.00	Non & mild frailty (1–5)	1.00	Low risk frailty (< 5)	1.00
	Mild frailty (4–5)**	1.18 (1.04 – 1.36) *p* = 0.013	Moderate frailty (6)**	1.17 (1.06 – 1.31) *p* = 0.003	Intermediate risk frailty (15–15)**	1.19 (1.08 – 1.32) *p* = 0.001
	Moderate frailty (6)	1.34 (1.15 – 1.55)	Severe frailty (7–9)	1.64 (1.46 – 1.84)	High risk frailty (> 15)**	1.19 (1.05 – 1.36) *p* = 0.009
	Severe frailty (7–9)	1.87 (1.60 – 2.19)				

^*^This logistic regression was adjusted for important baseline characteristics which were: 1) age, 2) sex, 3) CCI, 4) NEWS-2, and 5) DPS

^**^This indicates that the p-value is not significant at *p* < 0.001 level

When the CFS non-frail and mild frailty categories were combined together, individuals with CFS moderate and severe frailty, when compared non-frail individuals, had significantly higher 30-day mortality rate, LOS > 10 day, and 30-day readmission rate (supplementary table 6). After adjusting for covariates, 30-day mortality was still significantly higher for individuals with moderate and severe frailty (OR (95% CI), 1.36 (1.18 – 1.56) and 2.26 (1.96 – 2.61), respectively) (Table 4). The same is also true for LOS > 10 days (OR (95% CI), 1.39 (1.25 – 1.53) and 1.36 (1.21 – 1.53), respectively). However, 30-day readmission was significantly higher only for individuals with severe frailty (OR (95% CI), 1.64 (1.46 – 1.84)) but not for those with moderate frailty (OR (95% CI), 1.17 (1.06 – 1.31) (*p* = 0.003) (Table 4).

#### Hospital frailty risk score and outcomes

For the HFRS, individuals with intermediate and high risk frailty, compared to individuals with low risk frailty, had significantly higher 30-day mortality rate, LOS > 10 days, and 30-day readmission rate (supplementary table 6). The trends were similar after adjustment on covariates for 30-day mortality (OR (95% CI), 1.72 (1.49 – 1.99) and 2.16 (1.82 – 2.56), respectively) and LOS > 10 days (OR (95% CI), 2.73 (2.44 – 3.06) and 7.68 (6.71 – 8.80), respectively), but not for 30-day readmission which was not significantly higher for individuals with either intermediate (OR (95%CI), 1.19 (1.08 – 1.32)) (*p* = 0.001) or high risk of frailty (OR (95%CI), 1.19 (1.05 – 1.36)) (*p* = 0.009) (Table 4).

Overall, the CFS and HFRS were shown to be predictors for adverse outcomes, except that the HFRS was not a predictor for 30-day-readmission. The CFS was stronger than the HFRS in predicting 30-day mortality even after combining CFS non-frail and mild frailty categories, however, the HFRS was much stronger in predicting Length of in-hospital stay.

## Discussion

To our knowledge, this is the first study to compare and contrast the CFS and HFRS in the ED setting. Our kappa analyses showed that the CFS and HFRS identified different individuals by their frailty state, which resulted in low to slight agreement between the categories of both score. The adjusted logistic regression analyses showed that the ED-CFS and HFRS are predictors of adverse outcomes. Our findings indicated that each frailty score identified different populations who were at risk adverse hospital-related outcomes; highlighting potential benefits of collectively using both scores in the ED setting to guide clinical-decision making.

### Comparison with other studies

#### Correlation and level of agreement

Recent evidence compared the electronic Frailty Index, which has been developed in primary care settings, with the HFRS and showed a low to slight agreement between the two scores in identifying individuals across frailty strata [19]. This finding is similar to our findings when comparing the CFS and HFRS; indicating that different frailty scores identify different individuals across frailty strata.

With regards to the CFS and HFRS, both frailty tools seems to measure different aspects of frailty. The CFS is more based on clinical judgement, on patient appearance, and mobility capacity at the moment of clinical evaluation. Thus, functional aspects of frailty are perhaps emphasised when using this scale. Conversely, the HFRS scale is basically derived from ICD-10 codes. Thus, it is more grounded on the presence of multimorbidity and complex multimorbidity and less centered on the functional aspects of patients. This might explain why the correlation between the two frailty tools, based on the findings from our study, is low. However, both scales were shown in our study to be able to predict adverse outcomes, because both frailty and multimorbidity are associated with adverse outcomes and show a consistent overlap in many individuals. The point is that the HFRS is automated and so allows all patients (or nearly all) to be tracked throughout the system, to look at service level outcomes. The CFS is more clinically oriented, but often incomplete, so does not allow population level tracking.

#### Hospital frailty risk score

Since its introduction in 2018, the HFRS has been validated in several Western countries [13, 19–31] (supplementary table 7). Our findings showed that the HFRS is a predictor for mortality and LOS which are consistent with the findings of previous studies [13, 19–31] (supplementary table 7). However, we found that the HFRS is not a predictor for 30-day readmission and the evidence around the risk of unplanned readmission from previous studies is by far less consistent [13, 19–31] (supplementary table 7).

#### Clinical frailty scale in emergency care

For the CFS at ED triage, our findings were consistent with other studies assessing the ability of the CFS at the ED to predict hospital-related outcomes. Previous literature showed that the ED-CFS is a predictor for mortality [14, 32, 33] and LOS [33]. However, there were inconsistent findings around readmission [14, 33, 34] (supplementary table 8). The available evidence from the UK investigating the predictive ability of the ED-CFS showed that the ED-CFS is not a predictor of 30-day readmission [33] and the probability of readmission by two years increased with increasing frailty up until CFS 6 (moderate frailty), but then decreased (CFS 9 had the lowest rate of being readmitted) [14] (supplementary table 8). However, our findings showed that the CFS at the ED is a predictor of 30-day readmission and the odds of readmission increased with increasing frailty severity, which is consistent with a recent evidence from the United States of America [34] (supplementary table 8).

### Strengths and limitations

This study is the first study to compare and contrast the level of agreement between the CFS and HFRS. We used data from one of the largest hospitals in the UK which embedded risk stratification for frailty in the ED since 2016. We also adjusted our analyses for both statistically and clinically significant covariates. No bias was evident from our assessment of baseline characteristics in Table 1 of this study. However, there were few limitations that need to be highlighted. First, as our study was based on routinely collected data, which means that they depend on routine clinical practice [35]; not all individuals were assessed for frailty using the CFS despite that the CFS has been embedded in the ED for several years. This could introduce a risk of bias which may impair generalisation as the results of this study were only derived from a section of the frail population.

As the aim of our study is to assess the correlation between and the ability of the CFS and HFRS, we included all frailty and/or frailty risk scores resulting from the CFS and HFRS. We knew, for example, that the population of CFS 9 may differ substantially from the population and skew the results. However, excluding this population from our study could result in misleading findings (correlation and predictive ability findings) as we have patients with lower CFS scores (CFS < 9), but high HFRS scores (HFRS > 15); possibly leading to high risk of selection bias.

We only included patients who assigned CFS score at the ED and then admitted to hospital to calculate their HFRS based on ICD-10 codes which are calculated only if patients are admitted to hospital. This could introduce a risk of selection bias. It also prevented us to assess whether CFS and/or HFRS were associated with the decision to admit the patient to regular wards or not. Therefore, future research is needed to assess if the CFS collected at the ED and historic HFRS could aid in decision-making for admitting patients to hospital or not.

We calculated our HFRS data based on ICD-10 codes from index admission alone, which represents a limitation of our findings. A recent study showed that using the current admission alone to construct the HFRS could predict increased LOS and in-hospital mortality, but not 30-day readmission [36]. However, it recommended constructing the HFRS from current admission and from the previous two admissions within the last two years prior to current admission as this HFRS data was shown to be a powerful predictor for lengthier in-hospital stay and in-hospital mortality, but it was less predictive for emergency readmission [36].

The retrospective nature represents a limitation in this study. Finally, data was collected from a single-centre which may limit the applicability of our findings to the UK population.

The findings from this study highlight the need to obtain more frailty information in the ED as different frailty scores identified different population who were at risk of adverse hospital-related outcomes. They also suggest the need for future studies assessing the use of historic HFRS and other validated frailty scores to guide frailty identification and assessment in the ED.

## Conclusion

The CFS and HFRS had low/slight agreement, which indicates that the scores identify different levels of frailty severity when used in the same population. With both scores, the risk of adverse of hospital-related outcomes increased as frailty risk/severity increased as the CFS and HFRS were predictors for 30-day mortality and LOS > 10 days, however, the CFS was the only predictor for 30-day readmission after adjustment for baseline covariates. Further research assessing the use of historic HFRS and other validated frailty scores in the ED is needed to appropriately guide clinical decision-making for the older adults.

## Supplementary Information

**Additional file 1: Supplementary table 1. **Information about important study basline covariates. **Supplementary table 2.** Two way cross-tabulation of CFS and HFRS categories — original categories. **Supplementary table 3.** Two way cross-tabulation of CFS and HFRS categories — frail versus non-frail categories. **Supplementary table 4.** Hospital-related outcomes by CFS, HFRS categories – mean (SD) and frequencies (%). **Supplementary table 5.** Adjusted logistic regression for study covariates in the CFS and HFRS multivariate logistic regression models. **Supplementary table 6.** Univariate logistic regression for the CFS and HFRS. **Supplementary table 7.** Available studies assessing the ability of the HFRS to predict hospital-related outcomes (mortality, LOS, and readmission). **Supplementary table 8.** Previous studies assessing the ability of the ED-CFS to predict hospital-related outcomes.

## Data Availability

The datasets used and/or analysed during the current study are available from the corresponding author on reasonable request.

## Abbreviations

ED: Emergency Department
LOS: Length of Stay
CFS: Clinical Frailty Score
UK: United Kingdom
HFRS: Hospital Frailty Risk Score
ICD–10: International Classification of Diseases 10^th^ edition
LRI: Leicester Royal Infirmary
NEWS: National Early Warning Score
DPS: Dynamic Priority Score
CCI: Charlson Comorbidity Index
SD: Standard Deviation
OR: Odd Ratio
CI: Confidence Interval
