# Supplementary material for: A comparison between the clinical frailty scale and the hospital frailty risk score to risk stratify older people with emergency care needs
Source: BMC Emerg Med. 2022 Oct 25;22:171. doi: 10.1186/s12873-022-00730-5 (PMC9598033; doi:10.1186/s12873-022-00730-5)
Supplement: Supplementary file 1 — Additional file 1: Supplementary table 1. Information about important study basline covariates. Supplementary table 2. Two way cross-tabulation of CFS and HFRS categories — original categories. Supplementary table 3. Two way cross-tabulation of CFS and HFRS categories — frail versus non-frail categories. Supplementary table 4. Hospital-related outcomes by CFS, HFRS categories – mean (SD) and frequencies (%). Supplementary table 5. Adjusted logistic regression for study covariates in the CFS and HFRS multivariate logistic regression models. Supplementary table 6. Univariate logistic regression for the CFS and HFRS. Supplementary table 7. Available studies assessing the ability of the HFRS to predict hospital-related outcomes (mortality, LOS, and readmission). Supplementary table 8. Previous studies assessing the ability of the ED-CFS to predict hospital-related outcomes. [file 12873_2022_730_MOESM1_ESM.docx]

*Supplementary table 1 Information about important study basline covariates.*

| **Baseline Covariates** | **Description** |
| --- | --- |
| **National Early Warning Score-2 (NEWS-2)** | The NEWS-2 is the latest version of the National Early Warning Score (NEWS) which was produced in 2012 and then updated in December 2017 [1].  This score was generated to advocate the healthcare system to apply a standardised assessment and response to acute illness [1].  It was shown to be good in discriminating the risks of adverse outcomes just as the best existing systems and better than most [2].  When the recommended trigger threshold of the NEWS for urgent clinical response was applied (i.e., NEWS ≥5), the NEWS was found to be more sensitive and specific than most other existing systems [2].  The NEWS is based on an aggregate scoring system in which a score is allocated to physiological measurement which are already recorded in routine practice for individuals who are present or being monitored at hospital [1].  The basis of this scoring system is based on six physiological parameters: A) respiration rate, B) oxygen saturation, C) systolic blood pressure, D) pulse pressure, E) level of consciousness or new confusion, and F) temperature [1].  The NEWS-2 includes the six physiological parameters, plus a weighting score for supplemental oxygen [1]. |
| **Dynamic Priority Score (DPS)** | The DPS is a triage tool that is applied upon arrival at the ED to assess which individuals need more urgent care [3]. This was applied as a response to raised concerned in late 2015 in LRI hospital about the timeliness in which individuals arriving to the department by ambulance were assessed and establish their level of priority [3]. When individuals arrive to hospital by ambulance, they are either go to the resuscitation area if the individuals are classified as having life-threatening conditions or to the assessment area where the ambulance crew hand over the individuals to the nurse in charge and healthcare providers (paramedics, registrars, or danced nurse practitioners) working in the assessment area will establish their priority using DPS [3]. Four levels of priority can be generated by the DPS: A) immediate resuscitation, B) standard, C) urgent, and D) very urgent. |
| **Charlson Comorbidity Index (CCI)** | The CCI is a weighted index that was developed to predict death within one year of hospitalisation for individuals with specific comorbid conditions [4]. The CCI was generated by including nineteen conditions and each of these conditions was assigned to a scale from one to six, based one year mortality hazard ratio from a Cox proportional hazards model [4]. The resulted weighted were summed together to produce the CCI [4]. In 1992, Deyo and colleagues [5] separately adapted the CCI to ICD-9-CM diagnosis so that the CCI could be calculated using administrative data. At LRI, patient administration systems data contain a list of ICD-10-CM (used in this context for reimbursement), which were used to calculate the CCI score for individuals admitted at their index ED presentation. The CCI is categorised by some studies [6] into the following categories:   1. Mild (CCI 1-2) 2. Moderate (CCI 3–5) 3. Severe (CCI ≥6)   We used this categorisation in the descriptive and logistic regression analyses. |

1. Royal College of Physicians: **National Early Warning Score (NEWS) 2**. In*.* London, England: Royal College of Physicians; 2017.

2. Smith GB, Prytherch DR, Meredith P, Schmidt PE, Featherstone PI: **The ability of the National Early Warning Score (NEWS) to discriminate patients at risk of early cardiac arrest, unanticipated intensive care unit admission, and death**. *Resuscitation* 2013, **84**(4):465-470.

3. University Hospitals of Leicester NHS Trust: **Leicester Royal Infirmary Quality Report**. In*.* Leicester, England: University Hospitals of Leicester NHS Trust; 2016.

4. Charlson ME, Pompei P, Ales KL, MacKenzie CR: **A new method of classifying prognostic comorbidity in longitudinal studies: development and validation**. *Journal of chronic diseases* 1987, **40**(5):373-383.

5. Deyo RA, Cherkin DC, Ciol MA: **Adapting a clinical comorbidity index for use with ICD-9-CM administrative databases**. *Journal of clinical epidemiology* 1992, **45**(6):613-619.

6. Elliott A, Taub N, Banerjee J, Aijaz F, Jones W, Teece L, van Oppen J, Conroy S: **Does the clinical frailty scale at triage predict outcomes from emergency care for older people?** *Annals of Emergency Medicine* 2021, **77**(6):620-627.

Supplementary table 2 Two way cross-tabulation of CFS and HFRS categories — original categories.

|  | | **CFS** | | | | **Total** |
| --- | --- | --- | --- | --- | --- | --- |
|  |  | **Non- frail**  **(1-3)** | **Mild**  **(4-5)** | **Moderate**  **(6)** | **Severe**  **(7-9)** |  |
| **HFRS** | **Low-risk (<5)** | 1,298 (10.6%) | 2,024 (16.5%) | 600 (4.9%) | 305 (2.5%) | 4,227 (34.5%) |
|  | **Intermediate-risk (5-15)** | 736 (6.0%) | 2,500 (20.4%) | 1,553 (12.7%) | 1,077 (8.8%) | 5,866  (47.9%) |
|  | **High-risk (>15)** | 105 (0.9%) | 671 (5.5%) | 786  (6.4%) | 582 (4.8%) | 2,144 (17.5%) |
| **Total** | | 2,139 (17.5%) | 5,195  (42.5%) | 2,939 (24.0%) | 1,964  (16.0%) | 12,237  (100%) |

Supplementary table 3 Two way cross-tabulation of CFS and HFRS categories — frail versus non-frail categories.

|  | | **CFS** | | **Total** |
| --- | --- | --- | --- | --- |
|  |  | **Non-frail (1-5)** | **Frail (6-9)** |  |
| **HFRS** | **Non-frail (<5)** | 3,322 (27.1%) | 905 (7.4%) | 4,227 (34.5%) |
|  | **Frail (= >5)** | 4,012 (32.8%) | 3,998 (32.7%) | 8,010 (65.5%) |
| **Total** | | 7,334 (59.9%) | 4,903 (40.1%) | 12,237 (100%) |

Supplementary table 4 Hospital-related outcomes by CFS, HFRS categories – mean (SD) and frequencies (%).

| **Outcome** | **Study population** | **CFS** | | | | **HFRS** | | |
| --- | --- | --- | --- | --- | --- | --- | --- | --- |
|  | **Overall**  **(n= 12,237)** | **Non-frail (1-3)**  **(n= 2,139)** | **Mild frailty (4-5)**  **(n= 5,195)** | **Moderate frailty (6)**  **(n= 2,939)** | **Severe frailty (7-9)**  **(n = 1,964)** | **Low risk frailty (<5)**  **(n= 4,227)** | **Intermediate risk frailty (5-15)**  **(n= 5,866)** | **High risk frailty (>15)**  **(n= 2,144)** |
| **30-day mortality** | | | | | | | | |
| **Recorded** | 12,237 (100%) | 2,139 (100%) | 5,195 (100%) | 2,939 (100%) | 1,964 (100%) | 4,227 (100%) | 5,866 (100%) | 2,144 (100%) |
| **N (%)** | 1,631 (13.3%) | 156 (7.3%) | 533 (10.3%) | 439 (14.9%) | 503  (25.6%) | 339 (8.0%) | 874 (14.9%) | 418 (19.5%) |
| **Mean (SD)** | 13.0 (8.9) | 14.2 (8.8) | 14.1 (8.8) | 13.0 (8.8) | 11.3 (8.8) | 13.1 (9.4) | 12.1 (8.7) | 14.7 (8.5) |
| **LOS >10 days** | | | | | | | | |
| **Recorded** | 12,183 (99.6%) | 2,132 (99.7%) | 5,169 (99.5%) | 2,926 (99.6%) | 1,956 (99.6%) | 4,223 (99.9%) | 5,836 (99.5%) | 2,124 (99.1%) |
| **N (%)** | 3,323  (27.3%) | 361  (16.9%) | 1,361 (26.3%) | 958 (32.7%) | 643  (32.9%) | 516  (12.2%) | 1,660 (28.4%) | 1,147 (54.0%) |
| **Mean (SD)** | 19.8 (12.3) | 20.4 (17.2) | 19.3 (10.7) | 20.1 (13.0) | 20.2 (11.2) | 16.7 (8.7) | 18.7 (11.4) | 23.0 (14.3) |
| **30-day readmission** | | | | | | | | |
| **Recorded** | 12,237 (100%) | 2,139 (100%) | 5,195 (100%) | 2,939 (100%) | 1,964 (100%) | 4,227 (100%) | 5,866 (100%) | 2,144 (100%) |
| **N (%)** | 3,160 (25.8%) | 416 (19.4%) | 1,220 (23.5%) | 804 (27.4%) | 720 (36.7%) | 938 (22.2%) | 1,604 (27.3%) | 618 (28.8%) |
| **Mean (SD)** | 12.7 (8.9) | 11.7 (9.1) | 13.3 (9.0) | 13.2 (8.8) | 11.7 (8.8) | 12.3 (9.0) | 12.3 (8.9) | 14.2 (8.7) |

*Supplementary table 5 Adjusted logistic regression for study covariates in the CFS and HFRS multivariate logistic regression models.*

| **Covariate** | **30-day mortality OR (95% CI)** | | | **LOS >10, OR (95% CI)** | | | **30- readmission, OR (95% CI)** | | |
| --- | --- | --- | --- | --- | --- | --- | --- | --- | --- |
|  | **By original CFS categories** | **By combined CFS non-frail and mild frailty** | **By HFRS categories** | **By original CFS categories** | **By combined CFS non and mild frailty** | **By HFRS categories** | **By original CFS categories** | **By combined CFS non and mild frailty** | **By HFRS categories** |
| **Age (by year)** | 1.04 (1.03 – 1.05) | 1.04 (1.03 – 1.05) | 1.04 (1.03 – 1.05) | 1.02 (1.01 – 1.02) | 1.02 (1.01 – 1.03) | 1.01 (1.00 – 1.01) p = 0.188 | 1.02 (1.01 – 1.02) | 1.02 (1.01 – 1.03) | 1.02 (1.01 – 1.03) |
| **Sex** | | | | | | | | | |
| Female | 1.0 | 1.0 | 1.0 | 1.0 | 1.0 | 1.0 | 1.0 | 1.0 | 1.0 |
| Male | 1.28 (1.14 – 1.44) | 1.27 (1.13 – 1.43) | 1.24 (1.11 – 1.39) | 0.89 (0.82 – 0.98) p = 0.012 | 0.88 (0.81 – 0.96) p = 0.004 | 0.90 (0.82 – 0.99) p = 0.024 | 1.22 (1.11 – 1.33) | 1.21 (1.11 – 1.32) | 1.19 (1.09 – 1.30) |
| **CCI** | | | | | | | | | |
| 0 | 1.0 | 1.0 | 1.0 | 1.0 | 1.0 | 1.0 | 1.0 | 1.0 | 1.0 |
| 1-2 | 1.25 (1.07 – 1.46) p = 0.005 | 1.26 (1.08 – 1.47) p = 0.003 | 1.25 (1.07 – 1.46) p = 0.004 | 1.42 (1.28 – 1.58) | 1.47 (1.32 – 1.63) | 1.19 (1.06 – 1.32) p = 0.002 | 1.03 (0.93 – 1.15) p = 0.571 | 1.04 (0.94 – 1.16) p = 0.430 | 1.06 (0.96 – 1.18) p = 0.251 |
| 3-5 | 1.98 (1.66 – 2.35) | 2.01 (1.69 – 2.39) | 1.88 (1.59 – 2.24) | 1.80 (1.58 – 2.04) | 1.87 (1.65 – 2.12) | 1.25 (1.09 – 1.42)  P = 0.001 | 1.37 (1.21 – 1.56) | 1.40 (1.23 – 1.59) | 1.42 (1.25 – 1.62) |
| ≥6 | 5.76 (4.56 – 7.27) | 5.86 (4.64 – 7.40) | 6.00 (4.75 – 7.57) | 2.29 (1.87 – 2.81) | 2.39 (1.95 – 2.92) | 1.99 (1.61 – 2.46) | 3.01 (2.46 – 3.67) | 3.06 (2.51 – 3.73) | 3.18 (2.61 – 3.88) |
| **EWS (by each score)** | 1.14 (1.11 – 1.16) | 1.14 (1.11 – 1.16) | 1.16 (1.13 – 1.18) | 1.01 (0.99 – 1.03) p = 0.215 | 1.01 (1.00 – 1.03) p = 0.113 | 1.02 (1.00 – 1.04) p = 0.040 | 1.09 (1.07 – 1.11) | 1.09 (1.07 – 1.11) | 1.10 (1.08 – 1.12) |
| **DPS** | | | | | | | | | |
| Standard and urgent | 1.0 | 1.0 | 1.0 | 1.0 | 1.0 | 1.0 | 1.0 | 1.0 | 1.0 |
| Very urgent | 1.33 (1.16 – 1.53) | 1.33 (1.16 – 1.53) | 1.36 (1.19 – 1.57) | 1.06 (0.97 – 1.18) p = 0.190 | 1.06 (0.96 – 1.17) p = 0.271 | 1.12 (1.01 – 1.25) p = 0.030 | 1.22 (1.10 – 1.35) | 1.21 (1.09 – 1.34) | 1.22 (1.10 – 1.35) |
| Immediate Resuscitation | 1.60 (1.35 – 1.89) | 1.59 (1.34 – 1.88) | 1.65 (1.40 – 1.95) | 0.87 (0.75 – 1.00) p = 0.056 | 0.86 (0.75 – 0.99) p = 0.031 | 0.88 (0.76 – 1.02) p = 0.090 | 1.28 (1.12 – 1.47) | 1.27 (1.11 – 1.46) p = 0.001 | 1.29 (1.13 – 1.48) |

| **Outcome** | **CFS categories (by original categories)** | **Crude OR (95% CI)** | **CFS categories (by combined non-frail and mild frailty categories)** | **Crude OR (95% CI)** | **HFRS categories (by original categories)** | **Crude OR (95% CI)** |
| --- | --- | --- | --- | --- | --- | --- |
| **30-day mortality** | Non-frail (1-3) | 1.00 | Non & mild frailty (1-5) | 1.00 | Low risk frailty (<5) | 1.00 |
|  | Mild frailty (4-5) | 1.45 (1.20 – 1.75) | Moderate frailty (6) | 1.69 (1.49 – 1.93) | Intermediate risk frailty (15-15) | 2.01 (1.76 – 2.29) |
|  | Moderate frailty (6) | 2.23 (1.84 – 2.70) | Severe frailty (7-9) | 3.32 (2,92 – 3.77) | High risk frailty (>15) | 2.78 (2.38 – 3.24) |
|  | Severe frailty (7-9) | 4.38 (3.61 – 5.30) |  |  |  |  |
| **LOS >10 days** | Non-frail (1-3) | 1.00 | Non & mild frailty (1-5) | 1.00 | Low risk frailty (<5) | 1.00 |
|  | Mild frailty (4-5) | 1.75 (1.54 – 1.99) | Moderate frailty (6) | 1.58 (1.44 – 1.73) | Intermediate risk frailty (15-15) | 2.86 (2.56 – 3.18) |
|  | Moderate frailty (6) | 2.39 (2.08 – 2.74) | Severe frailty (7-9) | 1.59 (1.42 – 1.77) | High risk frailty (>15) | 8.43 (7.44 – 9.56) |
|  | Severe frailty (7-9) | 2.40 (2.07 – 2.78) |  |  |  |  |
| **30-day emergency readmission** | Non-frail (1-3) | 1.00 | Non & mild frailty (1-5) | 1.00 | Low risk frailty (<5) | 1.00 |
|  | Mild frailty (4-5) | 1.27 (1.12 – 1.44) | Moderate frailty (6) | 1.31 (1.19 – 1.45) | Intermediate risk frailty (15-15) | 1.32 (1.20 – 1.45) |
|  | Moderate frailty (6) | 1.56 (1.36 – 1.78) | Severe frailty (7-9) | 2.02 (1.81 – 2.24) | High risk frailty (>15) | 1.42 (1.26 – 1.60) |
|  | Severe frailty (7-9) | 2.40 (2.08 – 2.76) |  |  |  |  |

*Supplementary table 6 Univariate logistic regression for the CFS and HFRS.*

*Supplementary table 7 Available studies assessing the ability of the HFRS to predict hospital-related outcomes (mortality, LOS, and readmission).*

| **Study** | **Characteristics** | **HFRS Proportions and Outcomes (categorical and/or continuous)** | | | | | | | | | | |
| --- | --- | --- | --- | --- | --- | --- | --- | --- | --- | --- | --- | --- |
|  |  | **proportions, %** | | **Outcomes** | | | | | | | | |
|  |  |  |  | **30-day mortality** | | | **LOS >10 days** | | | **30-day readmission** | | |
|  |  |  |  | **Unadjusted OR (95% CI)** | **Adjusted OR (95%CI)** | **c-statistic** | **Unadjusted OR (95% CI)** | **Adjusted OR (95%CI)** | **c-statistic** | **Unadjusted OR (95% CI)** | **Adjusted OR (95%CI)** | **c-statistic** |
| Eckart et al. 2019 [20] | **Country:** Switzerland | **Low:** 63.5% | | 1.00 | 1.00 | Unadjusted = 0.66  Adjusted= N/A | 1.00 | 1.00 | Unadjusted = 0.72  Adjusted= N/A | 1.00 | 1.00 | Unadjusted = 0.54  Adjusted= N/A |
|  | **Population:** Patients aged ≥75 years who were hospitalised as an emergency between October 2015 and April 2018 (Monocentric, Aarau SWI) | **Intermediate:** 33.5% | | 2.53 (2.09-3.06) | 2.65 (2.17-3.25) |  | 3.47 (2.99-4.02) | 3.66 (3.14-4.28) |  | 1.04 (0.88-1.24) | 1.04 (0.87-1.24) |  |
|  | **Number of patients:** 4,957 | **High:** 2.9% | | 4.40 (2.94-6.57) | 4.83 (3.17-7.37) |  | 9.21 (6.51-13.01) | 9.75 (6.83-13.92) |  | 1.47 (0.95-2.26) | 1.67 (1.08-2.59) |  |
| Gilbert et al. 2018 [13] | **Country:** UK | **Low:** 42.4% | | 1.00 | 1.00 | Unadjusted = 0.60  Adjusted= 0.69 | 1.00 | 1.00 | Unadjusted = 0.68  Adjusted= 0.73 | 1.00 | 1.00 | Unadjusted = 0.56  Adjusted= 0.61 |
|  | **Population:** Patients aged ≥75 years or older hospitalised as an emergency between 1, April 2014 and 31, March 2015 (nationwide) | **Intermediate:** 37.6% | | 2.09 | 1.65 (1.62-1.68) |  | 3.36 | 3.29 (3.25-3.30) |  | 1.32 | 1.23 (1.22-1.25) |  |
|  | **Number of patients:** 1,023,337 | **High:** 20.0% | | 2.56 | 1.71 (1.68-1.75) |  | 6.03 | 6.01 (5.92-6.10) |  | 1.69 | 1.48 (1.46-1.50) |  |
| Gilbert et al. 2021 [18] | **Country:** France | **Low:** 45.4% | | 1.00 | 1.00 | Unadjusted = 0.62  Adjusted= 0.68 | 1.00 | 1.00 | Unadjusted = 0.67  Adjusted= 0.68 | 1.00 | 1.00 | Unadjusted = 0.57  Adjusted= 0.60 |
|  | **Population:** Patients aged ≥75 years who were hospitalised as an emergency in the year 2017 (nationwide) | **Intermediate:** 37.1% | | 1.79 (1.76-1.82) | 1.34 (1.32-1.37) |  | 2.56 (2.54-2.59) | 2.34 (2.32-2.37) |  | 1.24 (1.22-1.26) | 1.04 (1.02-1.05) |  |
|  | **Number of patients:** 1,042,234 | **High:** 17.5% | | 2.29 (2.25-2.34) | 1.38 (1.35-1.42) |  | 3.59 (3.55-3.64) | 3.27 (3.22-3.32) |  | 1.48 (1.45-1.51) | 1.00 (0.98-1.02) |  |
| Hollinghurst et al. 2021 [18] | **Country:** UK | **Low:** 53.6% | | NA | 1.00 | Unadjusted = N/A  Adjusted= 0.62 | NA | 1.00 | Unadjusted = N/A  Adjusted= 0.64 | NA | 1.00 | Unadjusted = N/A  Adjusted= 0.55 |
|  | **Population:** Patients aged ≥65 years who were hospitalised as an emergency between 2013-2017 (Wales, UK) | **Intermediate:** 33.5% | | NA | 1.34 (1.29-1.38) |  | NA | 1.68 (1.63-1.72) |  | NA | 1.31 (1.25-1.37) |  |
|  | **Number of patients:** 126,600 | **High:** 12.9% | | NA | 1.44 (1.37-1.51) |  | NA | 2.07 (1.99-2.14) |  | NA | 1.52 (1.43-1.61) |  |
| Kundi et al. 2019 [23] | **Country:** USA | **Acute Myocardial Infarction patients** | **Low:** 49.3% | NA | 1.00 | Unadjusted = N/A  Adjusted= 0.76 | NA | NA | N/A | NA | 1.00 | Unadjusted = N/A  Adjusted= 0.64 |
|  |  |  | **Intermediate:** 36.8% | NA | 3.31 (3.13-3.51) |  | NA | NA |  | NA | 1.90 (1.84-1.96) |  |
|  |  |  | **High:** 13.9% | NA | 3.59 (3.44-3.75) |  | NA | NA |  | NA | 2.98 (2.85-3.12) |  |
|  | **Population:** Patients aged ≥65 years who were hospitalised as an emergency due to acute myocardial infarction, heart failure, or pneumonia (nationwide) | **Heart Failure patients** | **Low:** 27.5% | NA | 1.00 | Unadjusted = N/A  Adjusted= 0.68 | NA | NA | N/A | NA | 1.00 | Unadjusted = N/A  Adjusted= 0.73 |
|  |  |  | **Intermediate:** 47.4% | NA | 2.80 (2.70-2.90) |  | NA | NA |  | NA | 1.57 (1.53-1.61) |  |
|  |  |  | **High:** 25.0% | NA | 3.53 (3.40-3.68) |  | NA | NA |  | NA | 2.91 (2.83-2.99) |  |
|  | **Number of patients:** 785,127 | **Pneumonia patients** | **Low:** 33.4% | NA | 1.00 | Unadjusted = N/A  Adjusted= 0.70 | NA | NA | N/A | NA | 1.00 | Unadjusted = N/A  Adjusted= 0.63 |
|  |  |  | **Intermediate:** 55.2% | NA | 2.08 (2.01-2.15) |  | NA | NA |  | NA | 1.81 (1.77-1.86) |  |
|  |  |  | **High:** 11.5% | NA | 2.45 (2.33-2.56) |  | NA | NA |  | NA | 2.82 (2.71-2.93) |  |
| McAlister et al. 2019 [27] | **Country:** Canada | **Low:** 74.1% | | NA | 1.0 | Unadjusted = N/A  Adjusted= 0.55 | NA |  | Unadjusted = N/A  Adjusted= 0.71 | NA |  | Unadjusted = N/A  Adjusted= 0.52 |
|  | **Population:** Patients aged ≥75 years with at least one urgent non-psychiatric hospitalisation between 2004 and 2010 (Ontario, Canada) | **Intermediate:** 23.3% | | NA | 1.39 (1.36–1.41) |  | NA | 3.66 (3.60–3.71) |  | NA | 0.84 (0.82–0.86) |  |
|  | **Number of patients:** 452,785 | **High:** 2.6% | | NA | 1.27 (1.20–1.33) |  | NA | 8.64 (8.30–8.99) |  | NA | 0.74 (0.69–0.79) |  |
| McAlister et al. 2020 [26] | **Country:** Canada | **Low:** 66.6% | | NA | NA | Adjusted without HFRS= 0.69  Adjusted with HFRS= 0.69 (idem) | NA | NA | Adjusted with HFRS for LOS >15 days= 0.71 | NA | NA | Adjusted without HFRS= 0.54  Adjusted with HFRS= 0.54 (idem) |
|  | **Population:** Patients aged ≥20 years who were hospitalised as an emergency due to heart failure between 2004-2016 | **Intermediate:** 26.4% | | NA | NA |  | NA | NA |  | NA | NA |  |
|  | **Number of patients:** 26,326 | **High:** 6.9% | | NA | NA |  | NA | NA |  | NA | NA |  |
| Kwok et al. 2020 [25] | **Country:** USA | **proportions, %** | | **In-hospital death** | | |  | | | | | |
|  |  |  |  | **Unadjusted OR (95% CI)** | **Adjusted OR (95%CI)** | **c-statistic** |  |  |  |  |  |  |
|  | **Population:** Patients aged ≥18 years who were hospitalised as an emergency due to heart failure between January 2004 and December 2014 (nationwide) | **Low:** 80.0% | | NA | 1.00 | NA |  |  |  |  |  |  |
|  |  | **Intermediate:** 19.9% | | NA | 2.28 (2.22-2.34) |  |  |  |  |  |  |  |
|  | **Number of patients:** 11,626,400 | **High:** 0.1% | | NA | 3.05 (2.57-3.62) |  |  |  |  |  |  |  |
| Kwok et al. 2019 [24] | **Country:** USA | **proportions, %** | | **In-hospital death** | | |  | | | | | |
|  |  |  |  | **Unadjusted OR (95% CI)** | **Adjusted OR (95%CI)** | **c-statistic** |  |  |  |  |  |  |
|  | **Population:** Patients aged ≥18 years who were hospitalised as an emergency with primary diagnosis of acute coronary Syndrome between January 2004 and December 2014 (nationwide) | **Low:** 86.5% | | NA | 1.0 | NA |  |  |  |  |  |  |
|  |  | **Intermediate:** 13.4% | | NA | 3.73 (3.66-3.80) |  |  |  |  |  |  |  |
|  | **Number of patients:** 7,398,572 | **High:** 0.1% | | NA | 2.57 (2.18-3.04) |  |  |  |  |  |  |  |
| Bonjour et al. 2021 [19] | **Country:** Switzerland | **proportions, %** | | **30-day survival** | | | **90-day survival** | | | **1-year survival** | | |
|  |  |  |  | **Unadjusted RR (95% CI)** | **Adjusted RR (95%CI)** | **c-statistic** | **Unadjusted RR (95% CI)** | **Adjusted RR (95%CI)** | **c-statistic** | **Unadjusted RR (95% CI)** | **Adjusted RR (95%CI)** | **c-statistic** |
|  | **Population:** Patients aged ≥65 years who were admitted to internal medicine ward between January 2009 and December 2017 (single centre) | **Low:** 36.7% | | NA | 1.00 | NA | NA | 1.0 | NA | NA | 1.0 | NA |
|  |  | **Intermediate:** 43.3% | | NA | 1.04 (0.95-1.14) |  | NA | 1.04 (0.97–1.12) |  | NA | 1.05 (1.00–1.11) |  |
|  | **Number of patients:** 22,323 | **High:** 20.0% | | NA | 1.31 (1.16-1.47) |  | NA | 1.22 (1.12–1.34) |  | NA | 1.21 (1.13–1.30) |  |
| Hannah et al. 2020 [22] | **Country:** USA | **proportions, %** | | **30-day readmission** | | |  | | | | | |
|  |  |  |  | **Unadjusted OR (95% CI)** | **Adjusted OR (95%CI)** | **c-statistic** |  |  |  |  |  |  |
|  | **Population:** Patients undergoing spine surgery between 1, January 2008 and 30, November 2016 (single center) | **Low:** 88.3% | | NA | NA | Adjusted without HFRS= 0.64  Adjusted with HFRS= 0.65 |  |  |  |  |  |  |
|  |  | **Intermediate:** 11.3% | | NA | NA |  |  |  |  |  |  |  |
|  | **Number of patients:** 11,754 | **High:** 0.4% | | NA | NA |  |  |  |  |  |  |  |
| Shebeshi et al. 2021 [29] | **Country:** Australia | **Proportions, %** | | **30-day in-patient mortality** | | | **LOS >10 days** | | | **28-day readmission** | | |
|  |  |  |  | **Unadjusted HR (95% CI)** | **Adjusted HR (95%CI)** | **c-statistic** | **Unadjusted OR (95% CI)** | **Adjusted OR (95%CI)** | **c-statistic** | **Unadjusted HR (95% CI)** | **Adjusted HR (95%CI)** | **c-statistic** |
|  | **Population:** Women aged from 75 to 95 years from the ALSWH cohort and hospitalised as an emergency between 2001-2006 | **Low:** 77.1% | | NA | 1.00 | NA | 1.00 | 1.00 | NA | 1.00 | 1.00 | NA |
|  |  | **Intermediate:** 21.0% | | NA | 1.78 (1.47-2.17) |  | 2.97 /(2.37-3.73) | 2.86 (2.26-3.62) |  | 1.06 (0.80-1.41) | 1.06 (0.79-1.42) |  |
|  | **Number of patients:** 2,740 | **High:** 1.9% | | NA | 4.17 (2.00-8.66) |  | 4.47 (2.46-7.91) | 4.26 (2.32-7.63) |  | 0.74 (0.27-7.97) | 0.75 (0.28-2.03) |  |
| Bruno et al. 2019 [30] | **Country:** Germany | **HFRS score, mean (SD)** | | **Composite outcome (30-day in-patient mortality + readmissions within 30-days)** | | |  | | | | | |
|  |  |  |  | **Unadjusted HR (95% CI)** | **Adjusted HR (95%CI)** | **c-statistic** |  |  |  |  |  |  |
|  | **Population:** Patients aged ≥75 years who were hospitalised in the ICU (Monocentric, GER). | Mean (SD): 3.1 (3.3) | | NA | 1.09 (1.05-1.13) | NA |  |  |  |  |  |  |
|  | **Number of patients:** 1,498 |  |  |  |  |  |  |  |  |  |  |  |
| McAlister et al. 2019 [28] | **Country:** Canada | **HFRS Proportions (frail vs. non-frail), %** | | **Composite outcome (30-day in-patient mortality + readmissions within 30-days, frail vs non-frail for all patients** | | | **Composite outcome (30-day in-patient mortality + readmissions within 30-days, frail vs non-frail for patients aged ≥65 years** | | |  | | |
|  |  |  |  | **Unadjusted OR (95% CI)** | **Adjusted OR (95%CI)** | **c-statistic** | **Unadjusted OR (95% CI)** | **Adjusted OR (95%CI)** | **c-statistic** |  |  |  |
|  | **Population:** Patients discharged from two Edmonton hospitals in the years 2013-2014 | **Non-frail (0-5):** 56.2% | | NA | 1.0 | Unadjusted = N/A  Adjusted= 0.58 | NA | 1.0 | Unadjusted = N/A  Adjusted= 0.55 |  |  |  |
|  | **Number of patients:** 489 | **Frail >5:** 43.8% | | NA | 1.62 (0.95-2.75) |  | NA | 1.24 (0.58-2.63) |  |  |  |  |

*Supplementary table 8 Previous studies assessing the ability of the ED-CFS to predict hospital-related outcomes.*

| **Study** | **Country** | **Population** | **Number of patients** | **CFS proportions, %** | **Outcomes** | | | |
| --- | --- | --- | --- | --- | --- | --- | --- | --- |
| Elliott et al., 2021 [31] | UK | Individuals aged ≥65 yeas attending at single, centralised ED between 1, October 2017 and 30, September 2019). | 52,562 | CFS 1-3: 32.1%  CFS 4-5: 37.0%  CFS 6: 17.9%  CFS 7-8: 12.3%  CFS 9: 0.7% | **Fine and Gray regression comparison of time from arrival at ED to readmission by two years, with death as a competing risk.**  **Adjusted sub-distribution HR (95% CI)**  CFS 1-3: 1.0  CFS 4-5: 1.43 (1.35–1.50)  CFS 6: 1.56 (1.47–1.66)  CFS 7-8: 1.20 (1.12–1.29)  CFS 9: 0.50 (0.36-0.70) | **Cox proportional hazards regression comparison of time from arrival at ED to death by two years.**  **Adjusted HR (95% CI)**  CFS 1-3: 1.0  CFS 4-5: 1.71 (1.55–1.88)  CFS 6: 2.44 (2.21–2.69)  CFS 7-8: 3.65 (3.30–4.03)  CFS 9: 5.60 (4.59–6.82) |  |  |
| Kaeppeli et al., 2020 [32] | Switzerland | Individuals aged ≥65 years attending at the ED of a tertiary care centre during nine weeks period between 18, March 2019 and 20, May 2019. | 2,393 | CFS 1: 1.5%  CFS 2: 14.5%  CFS 3: 28.4%  CFS 4: 18.8%  CFS 5: 12.5%  CFS 6: 10.4%  CFS 7: 7.8%  CFS 8: 5.3%  CFS 9: 0.7% (excluded from analyses) | **Mortality**  **Adjusted HR (95% CI):**  CFS 1-4: 1.0  CFS 5: 4.04 (2.17-7.55)  CFS 6: 5.99 (3.32-10.82)  CFS 7-8: 13.4 (7.46-20.27) | **30-day mortality**  **Adjusted OR (95% CI):**  CFS 1-4: 1.0  CFS 5: 4.12 (2.15-7.82)  CFS 6: 6.24 (3.38-11.57)  CFS 7-8: 12.3 (8.07-22.85)  **AUC (95% CI):**  CFS: 0.81 (0.77-0.85) | **ICU admission**  **Adjusted OR (95% CI):**  CFS 1-4: 1.0  CFS 5: 1.22 (0.71–2.01)  CFS 6: 2.72 (1.74–4.19)  CFS 7-8: 3.73 (2.57–5.37)  **AUC (95% CI):**  CFS: 0.69 (0.66-0.73) | **Hospitalisation**  **Adjusted OR (95% CI):**  CFS 1-4: 1.0  CFS 5: 2.59 (1.92–3.51)  CFS 6: 2.61 (1.89–3.66)  CFS 7-8: 3.75 (2.73–5.25)  **AUC (95% CI):**  CFS: 0.72 (0.70-0.74) |
| Wallis et al., 2015 [33] | UK | Individuals aged ≥75 years who were admitted to the ED (all specialties) between 1, August 2013 and 31, July 2014. | 7,532 (5,764 with available CFS) | CFS 1: 1.5%  CFS 2: 5.8%  CFS 3: 18.2%  CFS 4: 17.8%  CFS 5: 16.3%  CFS 6: 22.1%  CFS 7: 14.2%  CFS 8: 3.0%  CFS 9: 1.2% | **In-patient death**  **Adjusted OR (95% CI):**  Continuous CFS: 1.60 (1.48-1.74) | **LOS ≥10 days**  **Adjusted OR (95% CI):**  Continuous CFS: 1.19 (1.14-1.23) | **Transfer to Geriatric Ward**  **Adjusted OR (95% CI):**  Continuous CFS: 1.33 (1.24-1.42) | **30-day readmission**  **Adjusted OR (95% CI):**  Continuous CFS: 1.04 (.0.99-1.10) |
| Serina et al., 2020 [34] | USA | Individuals aged ≥75 years enrolled at a U.S., urban, academic Level 1 GED from September 1, 2019 to January  31, 2020 | 8,258 | CFS 1: 7.2%  CFS 2: 16.3%  CFS 3: 26.8%  CFS 4: 14.7%  CFS 5: 7.8%  CFS 6: 7.8%  CFS 7: 5.9%  CFS 8: 1.7%  CFS 9: 0.2% | **Admission to hospital**  **Adjusted OR (95% CI):**  Continuous CFS: 1.42 (1.38-1.46)  **Hospital admission (optimal cut-point was CFS ≥4)**  **sensitivity and specificity, % (95% CI)**  CFS 4-9: Sensitivity 54.0% (52.4-70.1%) and specificity 70.1% (68.5-71.6) | **30-day readmission**  **Adjusted OR (95% CI):**  Continuous CFS: 1.29 (1.23-1.35) | **ED return visits within 9 days**  **Adjusted OR (95% CI):**  Continuous CFS: 1.01 (0.966-1.07)  **ED return visit (optimal cut-point was CFS ≥3)**  **sensitivity and specificity, % (95% CI)**  CFS 3-9: Sensitivity 78.8% (75.0-82.1%) and specificity 27.0% (26.0-28.1) |  |
